# Peripheral lymphocyte fluctuation as an indicator of severe immune‐related adverse events in patients treated with immune checkpoint inhibitors

**DOI:** 10.1002/cam4.5816

**Published:** 2023-03-19

**Authors:** Masafumi Haraguchi, Yasuhiko Nakao, Syouhei Narita, Kousuke Matsumoto, Masanori Fukushima, Ryu Sasaki, Takuya Honda, Satoshi Miuma, Hisamitsu Miyaaki, Kazuhiko Nakao

**Affiliations:** ^1^ Department of Gastroenterology and Hepatology Nagasaki University Graduate School of Biomedical Sciences Nagasaki Japan

**Keywords:** CTLA‐4 inhibitors, irAEs, liver injury, neutrophil‐lymphocyte ratio

## Abstract

**Aim:**

Immune checkpoint inhibitors (ICIs) have proven to be effective treatments for various cancers, but can also elicit immune‐related adverse events (irAEs). Given that severe irAEs can be life‐threatening, biomarkers that can predict the occurrence of irAEs are of paramount importance. ICIs affect the dynamics of lymphocytes, and alterations in these dynamics may play a role in the development and severity of irAEs. The aim of this study was to investigate the correlation between irAEs and changes in lymphocyte counts.

**Methods:**

Information on irAEs was collected from 226 ICI cases from 2014 to 2020. We compared lymphocyte counts before treatment and at the onset of irAE and investigated the association between lymphocyte count fluctuations and the presence and severity of irAE, the course after steroid treatment, and overall survival.

**Results:**

Of the 226 cases, 27 patients developed grade 3 or higher irAE. Compared to the other groups, the lymphocyte count in this group was significantly decreased at the time of irAE (*p* < 0.01). There was a trend toward a rapid increase in lymphocyte count in the steroid responder group compared to the non‐responder group. Regarding overall survival, patients with irAE had significantly longer survival than those without irAE (*p* = 0.0025). However, there was no association between changes in lymphocyte count and survival in patients with irAE.

**Conclusion:**

The percentage change in lymphocyte count was found to correlate with the incidence of severe irAEs. Close monitoring of the patient's condition is crucial when the lymphocyte count decreases during ICI treatment.

## 1 INTRODUCTION

The advent of immune checkpoint inhibitors (ICIs) has dramatically changed the treatment of malignant tumors. ICIs are considered as a standard treatment for carcinomas, as they have shown favorable therapeutic effects and prolonged survival rates.^1^, ^2^, ^3^, ^4^ Thus, cancer immunotherapy has made significant inroads into solid and hematological tumors, such as non‐small cell lung cancer,^1^, ^2^, ^5^ urothelial cancer,^6^ melanoma,^3^, ^4^ and refractory Hodgkin lymphoma,^7^ leading to a paradigm shift in cancer treatment. ICIs act on inhibitory receptors and ligands, which are immune checkpoint molecules, to block inhibitory signals,^8^ thereby releasing the brakes on the immune system and producing an anti‐tumor effect.

Although ICIs have an unprecedented mechanism of action as an immune‐mediated therapy, disadvantages, such as immune‐related adverse events (irAEs), cannot be ignored. Given the broad reach of the immune system within the body, irAEs can affect any organ^9^ and can lead to serious organ failure, which can affect prognosis.^10^, ^11^, ^12^ Therefore, predicting the onset of irAE‐related liver injury is an important issue for patients undergoing ICI treatment. However, it is difficult to predict the onset of irAEs based on clinical characteristics, such as sex, age, and duration of ICI use,^13^ which makes irAE management difficult. In addition, patients who develop irAEs have a better prognosis than those who do not develop them.^14^ This suggests that discontinuing ICI treatment due to irAEs may be difficult and that patients who respond well to treatment may develop multiple irAEs. As the number of patients receiving ICI therapy is expected to increase, clinicians will have more opportunities to treat patients with irAEs. As a result, the management of irAEs will become increasingly important.

Several studies have reported an association between irAEs and peripheral blood lymphocytes. Matsukane et al^15^ reported that the neutrophil‐lymphocyte ratio (NLR) was elevated at the onset of irAE, while Fujisawa et al^16^ reported that the lymphocyte counts were relatively higher in patients with grade 3/4 irAEs related to gastrointestinal and lung injury. However, the relationship between changes in peripheral blood lymphocyte counts and irAE grade and prognosis has not been fully investigated. Therefore, we aimed to investigate the relationship between irAEs and changes in lymphocyte count in patients admitted to our hospital.

## 2 METHODS

### 2.1 Patients and study design

Our study included 226 cases of 196 patients treated with ICIs at Nagasaki University Hospital between January 2014 and April 2020. We excluded patients who had an acute exacerbation, a history of corticosteroid or immunosuppressive treatment at the time of data collection that may have affected the laboratory data, obvious growth in a metastatic liver tumor, and lack of clinical information, such as evaluation of irAEs. The obtained data included age, sex, primary malignancy, ICI regimens, observation period, presence or absence of steroid treatment for irAEs, blood cell count, and biochemical tests. The presence or absence of irAE and irAE Grade were determined based on information obtained from the electronic medical record. The diagnosis of irAEs and the exclusion of diagnoses requiring differentiation were made by the treating physician, and information was obtained from the electronic medical record. The grade of irAE was defined according to the National Cancer Institute (NCI) Common Terminology Criteria for Adverse Events (CTCAE; version 5.0). This methodology was based on a previous report.^17^ For irAE‐related liver injury, the patterns of liver injury were defined based on previous reports as follows^18^, ^19^, ^20^: (i) hepatocellular pattern, alanine aminotransferase (ALT) level alone elevated ≥5fold above the upper limit of normal (ULN) or the ratio of serum activities (expressed as a multiple of ULN) of ALT and alkaline phosphatase (ALP) was ≥5; (ii) cholestatic pattern, ALP level alone elevated ≥2‐fold above the ULN or the ratio of serum activities of ALT and ALP  ≤ 2; (iii) mixed pattern, the ratio of the serum activities of ALT and ALP was >2 and <5, respectively. Each liver enzyme value was obtained from the electronic medical record.

For patients with irAEs, laboratory data obtained on the day of irAE onset were used for analysis. Lymphocyte counts at baseline were compared with those at the onset of irAEs. The percentage change in lymphocyte count was calculated as follows:

$$\%\text{change}=\left(\frac{\text{Value}\;\mathrm{at}\;\text{the onset of irAEs}}{\text{Value}\;\mathrm{at}\;\text{the start of treatment}}-1\right)\times 100$$

The neutrophil‐to‐lymphocyte ratio (NLR) was also calculated in all cases before each treatment and at the onset of irAEs. The percentage change in NLR was also performed as described above.

For patients without irAEs, lymphocyte counts or NLR at baseline were compared with those at 30 days posttreatment.

In addition, for patients who experienced irAEs and were treated with steroids, we also analyzed changes in lymphocyte counts over the course of steroid treatment. Lymphocyte counts at the start of steroid treatment were compared with those at days 3, 7, 14, and 28 after steroid treatment. The percent change in lymphocyte count was calculated as follows:

$$\%\text{change}=\left(\frac{\text{Value}\;\mathrm{at}\;N\;\text{days after treatment with steroids}}{\text{Value}\;\mathrm{at}\;\text{the start of treatment}}-1\right)\times 100$$

The study design was approved by the Nagasaki University Ethics Committee (approval number: 20051803). This study was conducted in accordance with the principles of the Declaration of Helsinki. We did not obtain formal consent from the patients because of the nature of the retrospective observational study. Instead, all patients were given the opportunity to opt out of the study.

### 2.2 Treatment protocol

Each patient with lung cancer received nivolumab 240 mg every 2 weeks (*n* = 34), pembrolizumab 200 mg every 3 weeks (*n* = 37), or atezolizumab 1200 mg every 3 weeks (*n* = 8). Each patient with renal cell carcinoma received nivolumab 240 mg every 2 weeks (*n* = 17), pembrolizumab 200 mg every 3 weeks (*n* = 32), nivolumab 240 mg, and ipilimumab 3 mg/kg every 3 weeks (*n* = 7). Each patient with melanoma received nivolumab 240 mg every 2 weeks (*n* = 20), pembrolizumab 2 mg/kg every 3 weeks (*n* = 7), ipilimumab 3 mg/kg every 3 weeks (*n* = 11), or nivolumab 80 mg and ipilimumab 3 mg/kg every 3 weeks (*n* = 3). Each patient with oral carcinoma received nivolumab 240 mg every 2 weeks (*n* = 26) or pembrolizumab 200 mg every 3 weeks (*n* = 1). In patients with gastric cancer, nivolumab 240 mg every 2 weeks (*n* = 21), and ipilimumab 3 mg/kg every 3 weeks (*n* = 2) was administered.

### 2.3 Statistical analysis

Data are presented as median (minimum‐maximum). Fisher's exact test was used to compare categorical data. Continuous variables were compared between groups using the Mann–Whitney U test or Kruskal–Wallis test. Multiple comparisons were performed using Dunnett's multiple comparison test or Fisher's exact test. Survival curves were assessed using the Kaplan–Meier method. Log‐rank test was used to compare survival curves. A landmark analysis at 3 or 4 months was also performed using log‐rank tests.^21^, ^22^ A *p* < 0.05 was considered statistically significant for all tests. Statistical analyses were performed using GraphPad Prism version 9 (GraphPad Software, Inc.) and JMP version 16 (SAS Institute Inc.).

## 3 RESULTS

### 3.1 Characteristics of cases with and without irAE

The 226 cases of immune‐related adverse events (irAEs) in 196 patients were classified as non‐irAEs, grade 1, grade 2, and grade 3 or 4 according to the presence or severity of irAEs, and the clinical characteristics of each group were subsequently compared. As shown in Table 1, 43.8% of all patients developed irAEs, and 11.9% had grade 3 or higher irAEs. There were no significant differences in age, gender, or carcinoma type between the groups. The duration and frequency of immunotherapy treatment did not differ between groups, but the proportion of patients in the severe irAE group receiving CTLA‐4 therapy was significantly higher than in the mild irAE group (*p* = 0.03). Blood laboratory values at baseline were also evaluated between groups. There were no differences in white blood cell count or neutrophil count between groups, but lymphocyte count tended to be higher in the grade 3 or 4 group compared to the other groups.

**TABLE 1:** Characteristics of cases with and without irAE.

Characteristics	Non irAE (*n* = 127)	Grade 1 (*n* = 32)	Grade 2 (*n* = 40)	Grade 3 or 4 (*n* = 27)	*p‐*value
Age (years)	71 (24–93)	75 (55–85)	69 (31–87)	71 (71–78)	0.33
Sex, male/female	92/35	25/7	28/12	19/8	0.79
Primary malignancy (NSCLC/urological cancer/melanoma/head and neck cancer/Gastric cancer)	38/28/23/19/19	13/8/6/4/1	17/13/5/3/2	9/6/8/1/3	0.42
ICI (anti‐PD1/anti‐PDL1/anti‐CTLA4/anti‐PD1 and anti‐CTLA4)	110/6/5/6	32/0/0/0	36/0/2/2	17/2/5/3	0.03
Number of each ICI administrations	5 (1–55)	5.5 (1–101)	6.5 (1–65)	3 (1–48)	0.18
Duration of ICI treatment (days)	78 (1–968)	89 (19–1814)	107 (22–1189)	54 (1–871)	0.52
Observation period (days)	395 (31–2240)	375 (62–2080)	529 (44–1852)	332 (58–1220)	0.23
Duration from the start of treatment to the onset of irAE (days)	–	65 (20–118)	99 (24–1062)	59 (6–210)	0.57
White blood cells at baseline (10^3/^μL)	6.1 (1.2–21)	6.1 (2.6–11.8)	6.6 (3–16.3)	5.8 (2.6–13)	0.48
Stab and seg cells at baseline (10^3/^μL)	3.9 (0.2–18.6)	3.8 (1.5–9.7)	4.1 (1.5–13.9)	3.4 (1.5–8.3)	0.75
Lymphocyte cells at baseline (10^3/^μL)	1.25 (0.3–3.9)	1.18 (0.5–2.2)	1.4 (0.7–2.7)	1.7 (0.8–6.4)	0.06
Eosinocyte cells at baseline (10^3/^μL)	0.14 (0–3.2)	0.16 (0–1.15)	0.17 (0.04–1.12)	0.19 (0.05–0.9)	0.24
Neutrophil‐lymphocyte ratio (NLR) at baseline	3.0 (0.2–23.5)	2.8 (1.3–12.2)	2.8 (1.0–10.3)	2.45 (0.8–4.7)	0.16
Total protein (g/dL)	6.8 (5.2–8.7)	7.1 (5.9–8.7)	7.1 (6.2–8.7)	7.2 (5.5–8)	0.18
Albumin (g/dL)	3.4 (1.5–4.8)	4 (2.8–4.6)	3.8 (1.6–4.3)	3.7 (2.7–4.7)	0.09
Total bilirubin at baseline (mg/dL)	0.5 (0.2–0.8)	0.5 (0.3–1.1)	0.45 (0.2–0.9)	0.6 (0.3–0.8)	0.50
AST at baseline (IU/L)	19 (6–35)	18 (10–38)	17 (9–44)	19 (10–34)	0.86
ALT at baseline (IU/L)	13 (4–27)	14 (4–25)	15 (6–43)	14 (8–28)	0.92
LDH (IU/L)	188 (103–1196)	191 (133–545)	183 (122–635)	210 (107–1048)	0.18
BUN at baseline (mg/dL)	16 (5–38)	19 (8–33)	14 (5–32)	17 (9–25)	0.10
Cr at baseline (mg/dL)	0.88 (0.04–2.91)	0.88 (0.59–2.43)	0.89 (0.48–2.91)	0.96 (0.54–1.66)	0.83
eGFR	64.8 (4.9–129.4)	62.8 (20.5–90.7)	64.7 (17.7–114)	57.6 (6.6–92.2)	0.49

*Note*: Data are shown as median (range) values.

Abbreviations: ALT, alanine transaminase; AST, aspartate aminotransferase; BUN, blood urea nitrogen; Cr, creatinine; CTLA‐4, cytotoxic T‐lymphocyte antigen‐4; eGFR, estimated glomerular filtration rate; ICI, immune checkpoint inhibitor; LDH, lactase dehydrogenase; NSCLC, non‐small cell lung cancer; PD‐1, programmed cell death protein 1; PD‐L1, programmed cell death ligand 1.

### 3.2 Association between the changes in lymphocyte count and grade of irAEs

**FIGURE 1:**
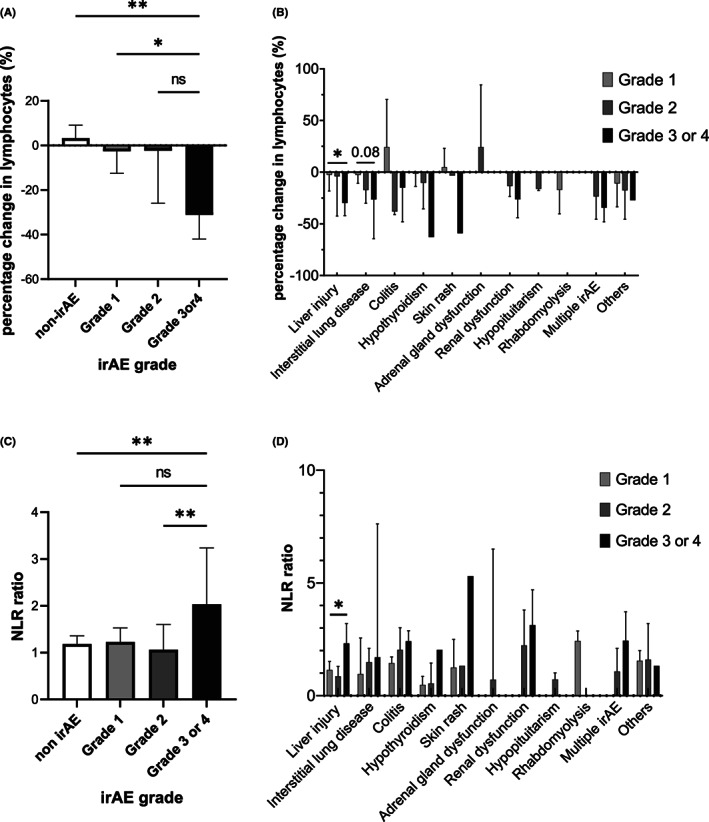
Comparison of the percentage change in lymphocyte count among the patients with or without irAEs. (A) The percentage change in lymphocyte count was significantly lower in the patients with grade 3 or 4 irAEs than in the non‐irAE group (*p* < 0.01) or in the grade 1 group (*p* = 0.02). (B) The percentage changes in lymphocyte count in various irAEs and association with irAE grade. The percentage changes in lymphocyte count in patients with grade 3 liver injury was significantly lower than those with grade 1 irAE (*p* < 0.05). (C) The NLR ratio was significantly higher in patients with grade 3 or 4 irAEs compared to those with non‐irAE or grade 2 (*p* < 0.01). (D) The NLR ratio in various irAEs and association with irAE grade. The NLR ratio in patients with grade 3 liver injury was significantly higher than those with grade 1 irAE (*p* < 0.05). *p < 0.05, **p < 0.01. irAEs, immune‐related adverse events; NLR, neutrophil‐to‐lymphocyte ratio; ns, not significant.

Next, we compared the percentage change in lymphocyte counts among the groups. As shown in Figure 1, the percentage change in lymphocyte count in grade 3 or 4 group was −31.1%, indicating that the percentage change in lymphocyte count in this group was significantly lower than that in the non‐irAE groups (*p* = 0.005, Figure 1A) or grade 1 group (*p* = 0.02). The cases of irAE were classified according to each affected organ (Table 2). A comparison of the percentage change in lymphocyte count for each organ by irAE grade showed that the rate of decrease in lymphocyte count tended to be more pronounced in grade 3 irAE cases than in the other groups at each site (Figure 1B). Additionally, the background factors contributing to the percentage change in lymphocyte count were also investigated using univariate analysis, but no significant factors were noted (Table 3).

**TABLE 2:** Classification of each irAE and grade.

Classification	Grade 1	Grade 2	Grades 3 or 4	Total
Liver injury (hepatocellular/mixed/cholestatic)	0/5/10 (42.8)	3/3/4 (28.6)	5/2/3 (28.6)	35
Interstitial lung disease (%)	9 (42.8)	6 (28.6)	6 (28.6)	21
Colitis (%)	5 (38.4)	5 (38.4)	3 (23.2)	13
Hypothyroidism (%)	2 (22.2)	6 (66.7)	1 (11.1)	9
Skin rash (%)	6 (75)	1 (12.5)	1 (12.5)	8
Adrenal gland dysfunction (%)	0	5 (100)	0	5
Renal dysfunction (%)	0	2 (50)	2 (50)	4
Hypopituitarism (%)	0	2 (100)	0	2
Rhabdomyolysis (%)	2 (100)	0	0	2
Multiple irAE (%)	0	4 (50)	4(50)	8
Others (%)	2 (25)	5 (62.5)	1 (12.5)	8

Abbreviation: irAE, immune‐related adverse events.

**TABLE 3:** Correlation between the low percentage changes in lymphocyte count and background factor.

Characteristics (values)	*p‐*value
Age (years) (median: 71)	0.28
Sex, male/female (146/50)	0.84
Primary malignancy: NSCLC/urological cancer/melanoma/head and neck cancer/gastric cancer (72/49/29/26/20)	0.18
ICI: anti‐PD1/anti‐PDL1/anti‐CTLA4/anti‐PD1 and anti‐CTLA4 (194/8/13/11)	0.14
History of ICI: 0/1/2/3/4 (196/21/8/1)	0.75
Number of each ICI administration (median: 5)	0.42
Duration of ICI treatment, days (median: 78)	0.48

Abbreviations: CTLA‐4, cytotoxic T‐lymphocyte antigen‐4; ICI, immune checkpoint inhibitor; NSCLC, non‐small cell lung cancer; PD‐1, programmed cell death protein 1; PD‐L1, programmed cell death ligand 1.

We also investigated the association between the percentage change in NLR values and any irAEs. Similar to lymphocyte count, the percentage change in NLR in grade 3 or 4 group was significantly higher than that in the other groups (grade 3 vs. non‐irAE; *p* = 0.003, grade 3 vs. grade 2; *p* = 0.01, Figure 1C). When compared by each organ, the NLR tended to be higher in the grade 3 group compared to the other groups (Figure 1D).

### 3.3 Association between treatment response to steroids and lymphocyte count fluctuations

**FIGURE 2:**
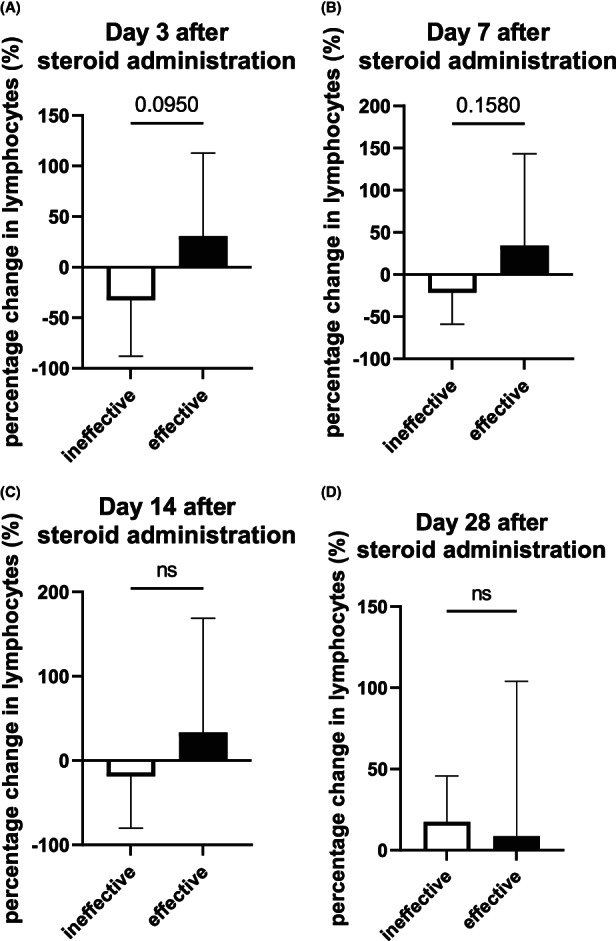
Comparison of the percentage change in lymphocyte cells among patients with irAEs treated with steroids. At day 3 after steroid administration, the percentage change in lymphocyte cells was higher in the patients who responded to steroid treatments (A). As days 7 to 28 progressed, the difference in percentage change in lymphocyte count between the two groups decreased (B–D). irAEs, immune‐related adverse events; ns, not significant.

We evaluated lymphocyte count fluctuations in patients with irAEs treated with steroids. In our study, 25 cases were treated with steroids for irAEs and two cases were excluded due to missing data. We evaluated the effect of steroid treatment based on the information in the electronic medical records and divided the patients into effective and ineffective treatment groups. As shown in Table 4, 19 patients showed a response to steroid therapy, with a mean time to onset of therapeutic effect of 7.58 ± 4.15 days. The lymphocyte counts at the start of steroid treatment were compared with those at days 3, 7, 14, and 28 after steroid treatment. The effective group tended to recover lymphocyte counts quickly after steroid administration, whereas the ineffective group showed a slower recovery (Figure 2A–D).

**TABLE 4:** Clinical characteristics of patients treated with steroids.

Characteristics	Values
Age (years)	72 (54–87)
Sex, male/female	15/8
Primary malignancy (NSCLC/urological cancer/melanoma/head and neck cancer/gastric cancer)	7/4/9/1/2
ICI (anti‐PD1/anti‐PDL1/anti‐CTLA4/anti‐PD1 and anti‐CTLA4)	12/6/2/3
History of ICI (2/3/4)	1/20/2
Number of ICI administrations	4 (1–24)
Duration of ICI treatment (day)	65 (1–871)
Observation period (day)	395 (58–1201)
White blood cell count	5400 (2600–12,200)
Stab and seg cell count	3300 (1570–8300)
Lymphocyte cell count	1540 (790–2910)
Effect of steroids on irAE (effective/ineffective)	19/4
Days required for symptom improvement	6 (2–21)

*Note*: Data are shown as median (range) values.

Abbreviations: CTLA‐4, cytotoxic T‐lymphocyte antigen‐4; ICI, immune checkpoint inhibitor; irAE, immune‐related adverse events; NSCLC, non‐small cell lung cancer; PD‐1, programmed cell death protein 1; PD‐L1, programmed cell death ligand 1.

### 3.4 Overall survival rate with or without irAE, by each grade, lymphocyte change rate, or NLR

**FIGURE 3:**
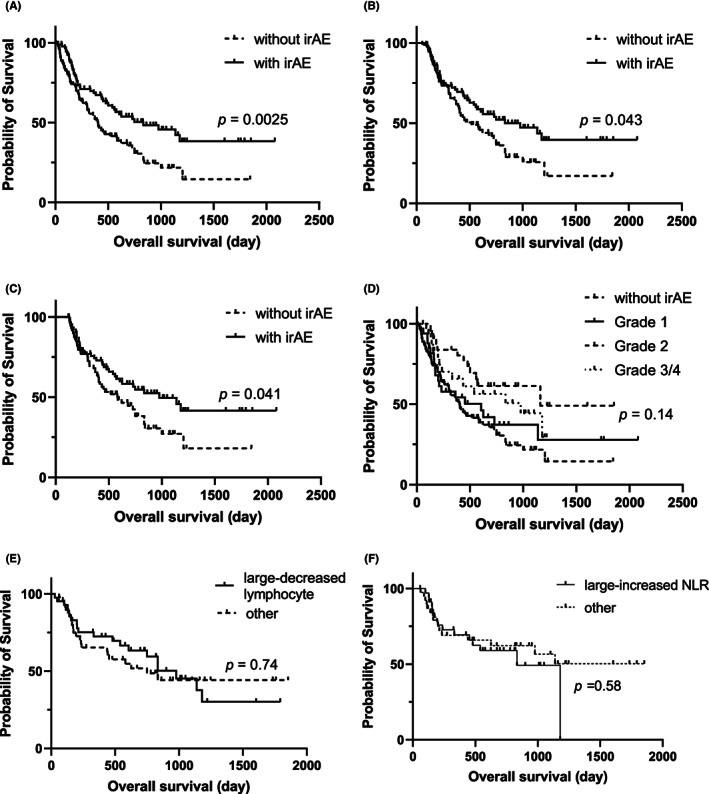
Overall survival rate with or without irAE, by each grade, lymphocyte change rate, or NLR. Patients with irAEs had significantly longer overall survival than those without irAEs (A). A landmark analysis was also performed at 3‐ (B) or 4 months (C) using log‐rank tests. The survival curves by landmark analysis also showed significantly longer survival in patients with irAEs (*p* = 0.043 and 0.041). There were no significant differences by irAE grade (D). In patients with irAE, there was no difference in overall survival according to the percentage change in lymphocyte count or the percentage change in NLR (E, F). irAEs, immune‐related adverse events; NLR, neutrophil‐to‐lymphocyte ratio.

Finally, we examined overall survival according to irAE grade. When comparing overall survival with and without irAEs, overall survival was significantly longer in the groups with irAEs than in the group without irAEs (*p* = 0.0025; Figure 3A). In addition, the survival curves in the groups stratified by the presence or absence of irAEs were also analyzed by landmark analysis. The landmark analysis survival curves also showed significantly longer survival in the irAE group (*p* = 0.043 and 0.041, respectively; Figure 3B,C). In contrast, there were no significant differences when subdivided by irAE grade (Figure 3D). The overall survival of patients with IrAE was also evaluated according to different rates of change in lymphocyte counts or in NLR. The patients with irAEs were divided into two groups, the large‐decrease and the other group, based on the median percentage change in lymphocyte counts (−12.8). The overall survival rates between the groups were compared and there was no significant difference in the overall survival between the groups. Similarly, there was no significant difference in overall survival between the two groups when grouped by the percentage change in NLR (Figure 3E,F).

## 4 DISCUSSION

In this study, we investigated the relationship between irAEs and the percentage change of lymphocyte count in patients receiving ICI treatment. Although patients with irAEs can have a severe course and should not be overlooked, it is not easy for physicians to diagnose a disease that is beyond their expertise. In addition, irAEs are unpredictable as to when and in which organ they will occur. Considering these issues, it is important to find a method to predict the onset of irAEs. Several studies have reported an association between peripheral lymphocyte counts and ICIs. It has been suggested that a decrease in lymphocytes in the peripheral blood might be associated with tumor reduction and gastrointestinal irAEs in patients treated with ICIs.^14^, ^17^ Conversely, previous reports have stated that an increase in lymphocyte counts was associated with improvement in OS,^17^, ^18^ and it can be stated that the relationship between ICI and lymphocyte counts has not yet been fully discussed. Based on various reports, we hypothesized that changes in peripheral blood lymphocyte counts might be useful in predicting irAEs.

In our study, the proportion of all patients with severe irAEs was consistent with previous reports.^17^, ^23^, ^24^ The proportion of patients who developed severe irAEs was higher in patients receiving CTLA‐4 treatment than in those who received other treatments as well as other reports,^2^, ^23^, ^25^ suggesting the frequency of irAEs might differ depending on the type of ICI. However, there was no statistically significant correlation between the incidence of irAEs and other clinical characteristics, such as duration of treatment or pretreatment blood test values, highlighting the challenge of predicting the incidence of irAEs in advance.

The group of patients with any grade 3/4 irAEs showed a significant decrease in lymphocyte count at the time of irAEs onset than other groups, suggesting that the percentage change in lymphocyte count might predict the development of severe irAEs. An approximately 30% decrease in lymphocyte count was observed in patients with grade 3 or higher irAEs. In a previous report,^16^ a lymphocyte count change rate of −23% was reported in patients with severe irAEs, which was similar to our study. We also investigated the correlation between background factors and the lower percentage change in lymphocyte cells. However, no background factors contributed to the percentage change in lymphocyte count, again suggesting the difficulty of predicting the development of irAE from background factors.

We also evaluated the NLR, which has been established as a prognostic marker for cancer patients and has been reported as a prognostic indicator for patients undergoing ICI therapy.^26^ In our study, the group with low NLR group had a better prognosis than the group with high NLR, as previously reported. However, pretreatment NLR was not a predictor of severe irAEs (data is not shown). Similarly, the pretreatment lymphocyte count was not a predictor of irAEs, highlighting the difficulty of predicting the development of irAEs based solely on pretreatment values. We also investigated the ratio of NLR prior to treatment to the onset of irAEs. The patients with severe irAEs were significantly higher values than those with mild irAEs, consistent with a previous report.^15^ This might have been related to a decrease in lymphocytes counts, as observed in our study. Additionally, Drobni et al^27^ reported the potential usefulness of monitoring changes in lymphocyte count in the study of irAE‐associated myocarditis. Similar to NLR, which is a simple and useful marker used in clinical settings, lymphocyte fluctuation is also expected to be monitored in the future.

We also examined the association between steroid treatment and lymphocyte count fluctuations. Although most of these adverse events can be managed by counteracting lymphocyte activation with steroids,^28^ steroid‐refractory/resistant immune‐related adverse events are seen in rare cases.^29^ At this time, no biomarkers have been reported to predict the efficacy of steroid treatment. In our study, there was a tendency for lymphocyte counts to recover a few days after steroid administration in patients who responded to steroids. Whether this tendency was due to steroid induction or discontinuation of ICI medication is unknown, but early recovery of lymphocyte counts might be predictive of improvement in irAEs. This issue should be reexamined with more cases receiving steroid treatment in the future. Regarding the underlying mechanism responsible for the effect of steroids on lymphocyte counts, the administration of steroid caused a decrease of lymphocyte counts that was maximal at 4–6 h and normalized by 24–48 h. This was predominantly the result of the redistribution of lymphocytes to bone marrow, spleen, thoracic duct, and lymph nodes.^30^, ^31^ Conversely, in the low‐to‐moderate dose range, the percentage of circulating T regulatory cells has been shown to increase in patients with sarcoidosis treated with intravenous methylprednisolone or prednisone, respectively.^32^, ^33^, ^34^ The mechanism by which steroids act on lymphocytes in patients with irAE remains unclear and deserves further study.

Concerning prognosis, the presence or absence of irAEs was also examined, and the results were consistent with those of previous reports,^13^, ^23^ with a favorable prognosis in the group that developed irAEs. There was no significant difference in the survival of the patients who developed irAEs among the different irAEs grades in our study, consistent with a previous report,^28^ suggesting the degree of irAEs does not correlate with prognosis. Rather, early response to irAEs and prevention of severe disease may prevent interruption of ICI and improve prognosis.

There were several limitations to our study that must be recognized. First, this was a single‐center, retrospective, cross‐sectional study; the causal relationship between irAEs and the percentage change of lymphocyte count remains unclear because of the cross‐sectional nature of this study. Therefore, caution should be exercised when interpreting our data. Additionally, 99 of 226 cases showed irAEs, of which only 27 cases showed severe irAEs. A long‐term or a multicenter study with more included cases should be conducted in the future. For the definition of some irAEs such as liver injury, colitis, and skin rash, pathological examination by biopsy was not performed, and only clinical judgment was performed. However, the same definition, as previously reported, was used in this study.^18^, ^19^, ^20^

Despite these limitations, our study has several notable strengths. To the best of our knowledge, this is the first study to evaluate the relationship between the degree of irAE‐related liver injury and lymphocyte count variation in peripheral blood. Predicting the likelihood of developing irAE‐related liver injury might lead to an early response and prevent severe irAEs. In addition, peripheral blood lymphocyte count data are readily available and inexpensive, and simple to interpret; therefore, it could be a useful biomarker.

## 5 CONCLUSIONS

Our study showed that a decrease in the percentage change in the lymphocyte count was associated with the development of severe irAEs. Therefore, when the lymphocyte count decreases during ICI administration, it is crucial to carefully monitor the patient's condition and approach treatment with the possibility of the development of irAEs.

## AUTHOR CONTRIBUTIONS

**Masafumi Haraguchi:** Conceptualization (lead); data curation (equal); formal analysis (equal); investigation (equal); project administration (lead); writing – original draft (lead); writing – review and editing (lead). **Yasuhiko Nakao:** Data curation (equal); investigation (equal). **Syouhei Narita:** Data curation (equal); investigation (equal). **Kousuke Matsumoto:** Data curation (equal); investigation (equal). **Masanori Fukushima:** Data curation (equal); investigation (equal); validation (equal). **Ryu Sasaki:** Conceptualization (supporting); data curation (equal); investigation (equal); validation (equal). **Takuya Honda:** Conceptualization (supporting); validation (supporting); writing – review and editing (supporting). **Satoshi Miuma:** Conceptualization (supporting); validation (supporting). **Hisamitsu Miyaaki:** Conceptualization (equal); validation (supporting); writing – original draft (supporting). **Kazuhiko Nakao:** Validation (supporting); writing – review and editing (supporting).

## FUNDING INFORMATION

This research did not receive any specific grant from funding agencies in the public, commercial, or not‐for‐profit sectors.

## ETHICS STATEMENT

The study design was approved by the Nagasaki University Ethics Committee (approval no. 20051803). This study was conducted in accordance with the principles of the Declaration of Helsinki. We did not obtain formal consent from the patients because of the nature of the retrospective observational study. Instead, we provided all patients with the opportunity to opt‐out.

## CONFLICT OF INTEREST STATEMENT

The authors declare that they have no conflict of interest.

## Data Availability

The data that support the findings of this study are available from the corresponding author upon reasonable request. The data are not publicly available due to privacy or ethical restrictions.
